# Discovery of caffeoylisocitric acid as a Keap1-dependent Nrf2 activator and its effects in mesangial cells under high glucose

**DOI:** 10.1080/14756366.2021.1998025

**Published:** 2021-12-11

**Authors:** Huankai Yao, Wenting Zhang, Feng Yang, Fengwei Ai, Dan Du, Yan Li

**Affiliations:** a Department of Microbial and Biochemical Pharmacy, School of Pharmacy & Jiangsu Key Laboratory of New Drug Research and Clinical Pharmacy, Xuzhou Medical University, Xuzhou, China; b Department of Laboratory Medicine, Xuzhou Center for Disease Control and Prevention, Xuzhou, China; c School of Stomatology, Xuzhou Medical University, Xuzhou, China

**Keywords:** Caffeoylisocitric acid, diabetic nephropathy, Nrf2 activator, mesangial cell

## Abstract

Diabetic nephropathy (DN) is one of the severe microvascular complications of diabetes mellitus. Oxidative stress resulting from aberrant metabolism of glucose mediates renal inflammation and fibrosis in the progression of DN. Nuclear factor erythroid 2-related factor 2 (Nrf2) is a transcription factor regulating the expression of antioxidant enzymes. Activating Nrf2 will give a promising therapy for DN. To discover novel Nrf2 activators, we have investigated caffeoylisocitric acid using mesangial cells under high glucose. The results showed at 10 μM, caffeoylisocitric acid significantly inhibited the self-limited proliferation of mesangial cells induced by high glucose. Further assessments have disclosed caffeoylisocitric acid mitigated oxidative stress, inflammation and accumulation of extracellular matrix resulting from high glucose via inactivating MAPK signalling. Meanwhile activation of Nrf2 was observed and involved in these effects through the interaction between Keap1 and caffeoylisocitric acid to disrupt Keap1-Nrf2 complex. Therefore, caffeoylisocitric acid is a promising Nrf2 activator targeting DN.

## 1. Introduction

As one of the most common microvascular complications of diabetes mellitus, diabetic nephropathy (DN) has been the leading cause of end-stage renal disease, which leads to the employment of dialysis and transplantation to improve renal failure^1^. Though the detailed pathogenesis of DN remains unclear, it is approved that oxidative stress resulting from aberrant metabolism of high glucose becomes the onset initiator^2^. Excessive reactive oxygen species (ROS) in the aberrant glucose metabolism can activate nuclear factor-kappa B (NF-κB) via activating mitogen-activated protein kinases (MAPK) signalling, which will enhance the expression of pro-inflammatory cytokines including tumour necrosis factor-alpha (TNF-α), interleukin-1 beta (IL-1β) and IL-6 and lead to the renal inflammation^3^. Meanwhile, ROS will induce the overexpression of transforming growth factor-beta 1 (TGF-β_1_) mediated by MAPK signalling^4^. In the progression of DN, TGF-β_1_ is the pivotal node since it can potently promote the secretion of extracellular matrix (ECM) proteins such as collagen IV, fibronectin and laminin directly or via activating connective tissue growth factor (CTGF)^5^. Under high glucose, accumulation of ECM together with the self-limited proliferation and hypertrophy of glomerular mesangial cells will result in the basement membrane thickening and glomerular mesangial expansion^6^. Meanwhile, high glucose can promote the podocyte-glomerular endothelial cell crosstalk to exacerbate the glomerular lesion via breaking the filtration barrier, since this interaction will result in endothelial cell dysfunction including increased ROS and inflammation as well as foot process effacement, apoptosis, and detachment of podocytes^7^. Without timely prevention, proteinuria and progressive renal function injury will be observed in clinic^8^. And then glomerulosclerosis and renal fibrosis occur until end-stage renal disease appears finally^9^. Therefore, targeting oxidative stress gives a promising approach for the treatment of DN^10^.

Nuclear factor erythroid 2-related factor 2 (Nrf2) is a transcription factor affording the expression of genes encoding antioxidant enzymes such as superoxide dismutase (SOD) and catalase (CAT)^11^. Under unstressed condition, Nrf2 is sequestered in cytosol by Kelch-like ECH-associated protein-1 (Keap1), which facilitates the ubiquitination of Nrf2 and degradation by 26S proteasome^12^. However, oxidants or electrophiles can disrupt the interaction between Keap1 and Nrf2 through interacting with cysteine residues in Keap1, which results in the dissociation of Keap1-Nrf2 complex and translocation of activated Nrf2 into nucleus from cytosol^13^. Then nuclear Nrf2 will bind to antioxidant response elements (ARE) in the promoter region of target genes to enhance their transcription^14^. Thus activation of Nrf2 can prevent oxidative stress-related diseases^15^. In the progression of DN, activation of Nrf2 ameliorates oxidative stress, inflammation and fibrosis resulting from high glucose^16^^,^^17^.

As the redox sensor, Keap1 is a member of BTB-Kelch family of proteins, which possesses a BTB domain at its N-terminus, an IVR domain and a Kelch domain at its C-terminus. And there are seven Neh domains (Neh1 to Neh7) in the sequence of Nrf2^18^. Structural analysis of Keap1-Nrf2 complex has shown two Keap1 monomers dimerise through respective BTB domains together with Cul3 and Rbx1 proteins, which made this complex itself function as an E3 ubiquitin ligase to facilitate ubiquitination of Nrf2^19^. Meanwhile, two Kelch domains of the homodimer recruit the DLG and ETGE motifs in Neh2 domain of Nrf2 to form the “hinge and latch” model since the affinity of DLG motif to Keap1 Kelch domain is much weaker that of ETGE^20^^,^^21^. The cysteine residues in both BTB and IVR domains can be modified by oxidants or electrophiles, which induces the structural changes of Kelch domain and leas to the dissociation of DLG motif from Kelch domain^22^. Therefore, direct targeting Kelch domain of Keap1 can also disrupt the interaction between Keap1 and Nrf2 and lead to the activation of Nrf2^23^.

**Figure 1.**
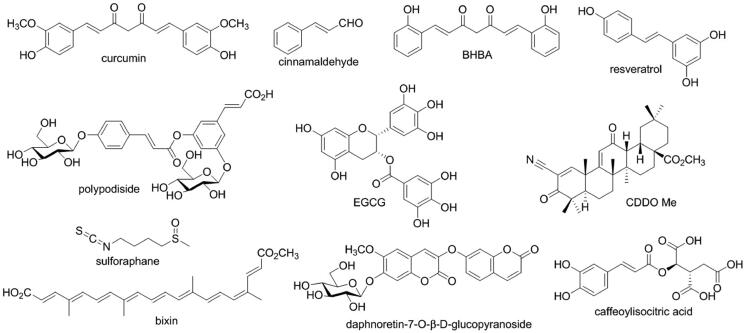
Nrf2 activators from natural products or their derivatives and caffeoylisocitric acid.

In the discovery of Nrf2 activators, natural products have become the pool of candidates. Some natural compounds such as curcumin, resveratrol, sulforaphane, and cinnamaldehyde showed significant activity to activate Nrf2^24^. Especially bardoxolone methyl (CDDO-ME), a synthetic derivative of oleananic acid, as an Nrf2 activator has been in phase 3 trial involving patients with DN. However, the trial was terminated due to the strong adverse effects related to safety^25^. In searching for Nrf2 activators 3-phenylpropanoic acid is an important fragment^26^, the moieties derived from which can also be found in many natural compounds (Figure 1). Our previous investigations have revealed polypodiside and daphnoretin-7-O-β-D-glucopyranoside as Nrf2 activators attenuated oxidative stress and accumulation of ECM mesangial cells under high glucose^27^^,^^28^, and both of them possess cinnamoyl group which is analogue of 3-phenylpropanoic acid. However, these compounds are dimers with glucose moiety, which results in the complex structures. To find novel Nrf2 activators, we have screened the natural compounds with cinnamoyl moiety in our group and caffeoylisocitric acid has been raised. As a rare cinnamic acid derivative, caffeoylisocitric acid is a condensed ester of a caffeoylic acid and an isocitric acid, which is first found in *Amaranthus cruentus*^29^. Recently, we have identified it from *Potentilla fragarioides* L^30^, whereas there is no report about its biological activity. Herein we report its effects on Nrf2 activation and certain activities associated with DN using cell-based assays.

## 2. Materials and methods

### 2.1. Chemicals and reagents

Caffeoylisocitric acid (CIA) was isolated from *Potentilla fragarioides* L^30^ with the purity of more than 98% analysed by HPLC. Cell counting kit-8 (CCK-8) assay kit was obtained from Dojindo Laboratories (Kumamoto, Japan). DyLight 594-conjugated secondary antibody was purchased from EarthOx Life Sciences (Millbrae, CA). ROS, malondialdehyde (MDA), SOD, and CAT assay kits, 4′,6-diamidino-2-phenylindole (DAPI) staining solution, rabbit IgG, nuclear and cytosolic protein extraction kit, bicinchoninic acid (BCA) protein assay kit, and enzyme-link chemiluminescence (ECL) assay kit together with TNF-α, IL-1β and IL-6 ELISA assay kits were supplied by Beyotime Biotechnology Institute (Shanghai, China). Bromodeoxyuridine (BrdU) cell proliferation assay kit was purchased from Shanghai Boyun Biotech (Shanghai, China). Human collagen IV, fibronectin, laminin, TGF-β_1_, CTGF, haem oxygenase 1 (HO-1), NAD(P)H:quinone oxidoreductase 1 (NQO1), glutathione S-transferase (GST) A1 and A2 ELISA kits were provided by Colorfulgene Biotechnology (Wuhan, China). ELISA kits for activated members of MAPK family including phosphorylated extracellular signal-regulated kinase 1/2 (p-ERK1/2), phosphorylated c-Jun N-terminal kinase (p-JNK), and phosphorylated p38 MAPK (p-p38 MAPK) were obtained from RayBiotech Life (Peachtree Corners, GA). The primary antibodies of Keap1, Nrf2, β-actin and lamin B1 together with horseradish peroxidase-conjugated secondary antibody were supplied by Abcam (Cambridge, UK). The ARE luciferase reporter plasmid (pGL4.37[luc2P/ARE/Hygro]), NF-κB response elements luciferase reporter plasmid (pGL4.32[luc2P/NF-κB-RE/Hygro]), renilla luciferase reporter plasmid (pRL-TK) and dual-luciferase reporter assay system were obtained from Promega (Madison, WI). Lipofectamine 2000 and the Nrf2 activator, sulforaphane (SFN), were furnished by Thermo Fisher Scientific (Waltham, MA). Negative control siRNA (NC-siRNA), Nrf2-siRNA and protein A agarose were provided by Santa Cruz Biotechnology (Dallas, TX).

### 2.2. Cell culture and treatment

Human glomerular mesangial cells were obtained from American Type Culture Collection (ATCC, Manassas, VA) and maintained in DMEM including 10% FBS, 100 U/mL penicillin and 100 mg/mL streptomycin under a humid condition with 5% CO_2_ and 95% air at 37 °C. The cells were divided into control group (Ctrl), high glucose group (HG), and experimental groups (caffeoylisocitric acid or sulforaphane). After starvation using serum-free medium for 24 h, the cells in high glucose group and experimental groups were exposed to high glucose (30 mM) for 24 h. At the same time, the cells in experimental groups were treated with certain caffeoylisocitric acid or sulforaphane. The cells in control group were incubated with DMEM together with normal glucose (5.6 mM).

### 2.3. Cck-8 assay

To assess the effects of caffeoylisocitric acid on the viability of human glomerular mesangial cells, CCK-8 assay was firstly implemented. In brief, the harvested cells were seeded in 96-well microplates and treated as above. Then CCK-8 solution (10 μL) was added. After incubation at 37 °C for 1 h, the absorbance was recorded on a microplate reader (Bio-Rad, Hercules, CA) at 450 nm. The cell viability was expressed as relative percentage of absorbance in experimental groups against that in control group.

### 2.4. Brdu incorporation assay

To detect the effects of caffeoylisocitric acid on the self-limited proliferation of mesangial cells under high glucose, we have performed BrdU incorporation assay. Briefly, the treated cells were incubated with 10 µL BrdU labelling solution in the assay kit at 37 °C for 3 h. After removing the solution, the cells were fixed with 200 µL FixDenat solution and incubated at room temperature for 30 min. Then the solution was removed and 100 µL anti-BrdU-POD working solution was added. After incubation for 90 min at room temperature, the cells were washed with PBS and then incubated with 100 µL substrate solution at room temperature for 30 min. Finally, the absorbance was read on the microplate reader at 370 nm.

### 2.5. Immunofluorescence assay

Immunofluorescence assay was employed to localise intracellular Nrf2. The glomerular mesangial cells were cultured in 12-well microplates with one coverslip in each well. After treated as above, the cells on the coverslips were washed with PBS and fixed with 4% paraformaldehyde at 4 °C for 15 min. Then PBS containing 0.1% Triton X-100 was used to permeabilise the cells for 10 min. And then the cells were blocked with 1% BSA for 30 min. Subsequently the cells were exposed to primary antibody against Nrf2 (1:200) and incubated at 4 °C overnight. After washed with PBS, the cells were incubated with DyLight 594-conjugated secondary antibody at 37 °C in the dark. Then the cells were washed with PBS again and stained with DAPI in the dark for 2 min. The coverslips were mounted on glass slides and the images were captured under a fluorescence microscope (Olympus, Tokyo, Japan).

### 2.6. Total and nuclear proteins extraction

Total and nuclear proteins were extracted using the RIPA lysis buffer solution or nuclear and cytosolic protein extraction kit in light of the supplier’s instructions. For the extraction of total proteins, the treated glomerular mesangial cells were harvested and lysed on ice with RIPA lysis buffer solution for 30 min. After centrifugation at 12,000 × g for 5 min, the supernatant was collected as total proteins after quantification using BCA protein assay kit. For the extraction of nuclear proteins, the treated cells were exposed to 200 µL buffer solution A containing 1 mM PMFS and votex was performed for 5 s. Then incubation was carried on ice for 15 min followed by the addition of 10 µL buffer solution B. After votex and incubation, centrifugation was implemented at 16,000 × g and 4 °C for 5 min. Then the supernatant was removed and the pallet was incubated with 50 µL nuclear protein extraction solution containing 1 mM PMSF on ice for 30 min. After vortex and centrifugation, the supernatant was collected as nuclear proteins for further analysis after determining the protein concentration using BCA protein assay kit.

### 2.7. Western blot analysis

Western blot was implemented to analyse Nrf2 in glomerular mesangial cells. In brief, the proteins were separated on 15% sodium dodecyl sulphate polyacrylamide gel electrophoresis (SDS-PAGE) and transferred to polyvinylidene fluoride (PVDF) membranes. After blocked with 5% non-fat milk at room temperature for 1 h, the membranes were incubated with specific primary antibodies including Nrf2 (1:1,000), β-actin (1:1,000) and lamin B1 (1:10,000) at 4 °C overnight. After washed with buffer solution containing 0.1% Tween-20 for three times, the membranes were incubated with horseradish peroxidase-conjugated secondary antibody (1:2,000) at room temperature for 2 h. The blots were visualised using ECL substrate and densitometric analysis was performed using ImageJ software (NIH, Bethesda, MD).

### 2.8. Dual luciferase reporter assay

The capacity of Nrf2 and NF-κB for transcriptional regulation in glomerular mesangial cells was evaluated using the dual luciferase reporter assay according to the supplier’s protocol. Briefly the glomerular mesangial cells were transfected with ARE luciferase reporter plasmid or NF-κB response elements luciferase reporter plasmid together with renilla luciferase reporter plasmid using lipofectamine 2000. After incubation at 37 °C for 24 h, the cells were treated as above. And then the luciferase activity was determined using dual luciferase reporter assay system on a Promega GloMax luminometer (Madison, WI). The relative transcription capacity of Nrf2 and NF-κB to respective response elements was derived from the normalised the firefly luciferase activity against the renilla luciferase.

### 2.9. Intracellular ROS assay

The intracellular ROS in glomerular mesangial cells was monitored using a ROS assay kit. Briefly, the treated cells were washed with serum-free medium for three times and incubated with 2′,7′-dicholorofluorescein diacetate (DCFH-DA) in the dark for 20 min at 37 °C. After washed with serum-free medium again, the fluorescence intensity was read on a fluorescence microplate reader (Molecular Devices, San Jose, CA) at excitation wavelength of 488 nm and emission wavelength of 525 nm. The ROS level was expressed as relative percentage of fluorescence intensity versus control group.

### 2.10. Mda content assay

The MDA content in glomerular mesangial cells was determined using the thiobarbituric acid method. In brief, the treated cells were lysed using ice-cold RIPA lysis buffer and centrifuged at 1,600 × g for 10 min. Then 100 µL supernatant was mixed with 200 µL working solution of the assay kit. After boiled for 15 min and cooled to room temperature, the sample centrifuged at 1,000 × g for 10 min and transferred to a 96-well microplate. The absorbance was read on a microplate reader at 532 nm. The MDA content was expressed as relative percentage of absorbance against that of control group.

### 2.11. Determination of SOD and CAT activity

To reveal the activity of antioxidant enzymes including SOD and CAT in glomerular mesangial cells, colorimetric method was chosen using the commercially available assay kits. Following the supplier’s instructions, the treated cells were lysed using ice-cold lysis buffer. After centrifugation at 12,000 × g for 5 min, the supernatant was sucked and quantified using a BCA protein assay kit. In SOD activity assay, the mixture including 20 µL supernatant, 160 µL tetrazolium/enzyme working solution and 20 µL initiating working solution was incubated at 37 °C for 30 min. The absorbance was read on a microplate reader at 560 nm. In the CAT activity assay, 10 µL supernatant, 10 µL H_2_O_2_ (250 mM) and 30 µL testing buffer solution were mixed and incubated at 25 °C for 5 min. After handled in light of the instruction, the absorbance was measured at 520 nm. The enzyme activity was presented as the relative percentage of absorbance in experimental groups versus that of control group.

### 2.12. Enzyme linked immunosorbent assay (ELISA)

The levels of proteins and cytokines related to fibrosis and inflammation in mesangial cells were detected through ELISA using the commercially available assay kits. For the secreted extracellular proteins, the treated cells were centrifuged at 1,000 × g for 20 min, and then the supernatant was collected for further analysis. For the intracellular proteins, the treated cells were lysed on ice and then centrifuged at 1,000 × g for 20 min to obtain the supernatant. After treated following the supplier’s instructions, the absorbance was recorded on a microplate reader at 450 nm. The levels of HO-1, NQO1, GST A1, GST A2, collagen IV, fibronectin, laminin, TGF-β_1_, CTGF, TNF-α, IL-1β, IL-6, p-ERK1/2, p-JNK and p-p38 MAPK were derived from the absorbance versus that of control group.

### 2.13. Si-RNA interference assay

To elucidate the role of Nrf2 in the effects of caffeoylisocitric acid on glomerular mesangial cells under high glucose, siRNA interference was employed. Briefly the cells were transfected with NC-siRNA or Nrf2-siRNA according to the supplier’s protocol, and western blot was performed to validate the successful transfection. Thereafter the cells were treated as above. And the levels of ROS, MDA, TNF-α, IL-1β, IL-6, collagen IV, fibronectin and laminin were determined on a microplate reader at 450 nm.

### 2.14. Co-immunoprecipitation assay

The interaction between Keap1 and Nrf2 in glomerular mesangial cells was explored through co-immunoprecepitation assay using protein A agarose. Following the supplier’s protocol, the treated glomerular mesangial cells were lysed on ice-cold lysis buffer and then centrifuged at 10,000 × g for 10 min at 4 °C. The supernatant was collected and preclear reaction was conducted with suspended protein A agarose to reduce the non-specific combination. After incubation at 4 °C for 30 min and subsequent centrifugation at 1,000 × g for 5 min, the supernatant was exposed to anti-Nrf2 or rabbit IgG at 4 °C for 1 h followed by addition of protein A agarose. After shaking at 4 °C overnight, the immunoprecipitates were centrifuged at 1,000 × g for 5 min. Then the supernatant was removed carefully and the pellets were washed with lysis buffer. Thereafter the pellets were suspended in SDS loading buffer and boiled for 5 min. The precipitated proteins were resolved by 15% SDS-PAGE and detected by western blot analysis with corresponding antibodies.

### 2.15. Molecular docking

Molecular docking was implemented using SYBYL X2.1 to disclose the detailed interaction between Keap1 and caffeoylisocitric acid. The 3D structure of caffeoylisocitric acid was generated from SYBYL sketch, which was optimised by Tripos force field and Gasteiger-Huckel charges. The x-ray structure of Keap1 Kelch domain was retrieved from RSCB Protein Data Bank (PDB code: 4IQK). After extracting the ligand and removing the water molecules in the structure, hydrogen atoms and charges were complemented. Then the protomol file was generated to establish the docking envelope and Surflex-Dock program with default parameters was operated for docking calculations.

### 2.16. Statistical analysis

All the experimental data were expressed as mean ± standard deviation analysed using GraphPad Prism 5.0 (San Diego, CA). One way analysis of variance (one way ANOVA) was performed and followed by Tukey test for multiple comparisons and Student’s t test for single comparisons. The *P* < 0.05 was considered significantly in statistics.

## 3. Results and discussion

### 3.1. Caffeoylisocitric acid reduces the survival of mesangial cells under high glucose

**Figure 2.**
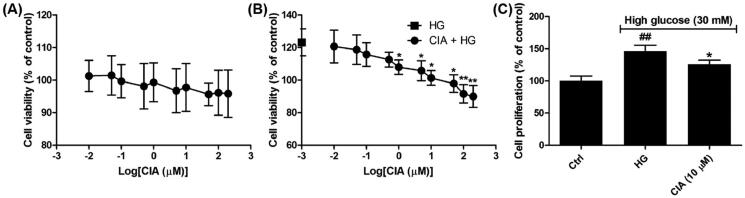
Effects of caffeoylisocitric acid on the survival of mesangiacl cells. (A) Viability of mesangial cells cultured in normal medium. (B) Viability of mesangial cells cultured in medium containging high glucose. (C) BrdU incorporation assay. *n* = 3, ^##^*P* < 0.01 *vs* control group, *P < 0.05 and **P < 0.01 *vs* high glucose group.

At the early stage of DN, high glucose has led to the self-limited proliferation of mesangial cells and accumulation of ECM, which collectively give rise to the thickening basement membrane and mesangial expansion^31^. As shown in Figure 2, though in the range of 0.01 to 200 µM caffeoylisocitric acid has no significant cytotoxicity on normal mesangial cells, it reduced the increasing cell viability induced by high glucose when the concentration is more than 1 µM. Further investigations using BrdU incorporation assay have revealed the self-limited proliferation of mesangial cells under high glucose was slightly inhibited by caffeoylisocitric acid at 10 µM (*P* < 0.05). These findings gave us the indication to explore caffeoylisocitric acid at 10 µM in the following experiments.

### 3.2. Caffeoylisocitric acid activates Nrf2 in mesangial cells under high glucose

**Figure 3.**
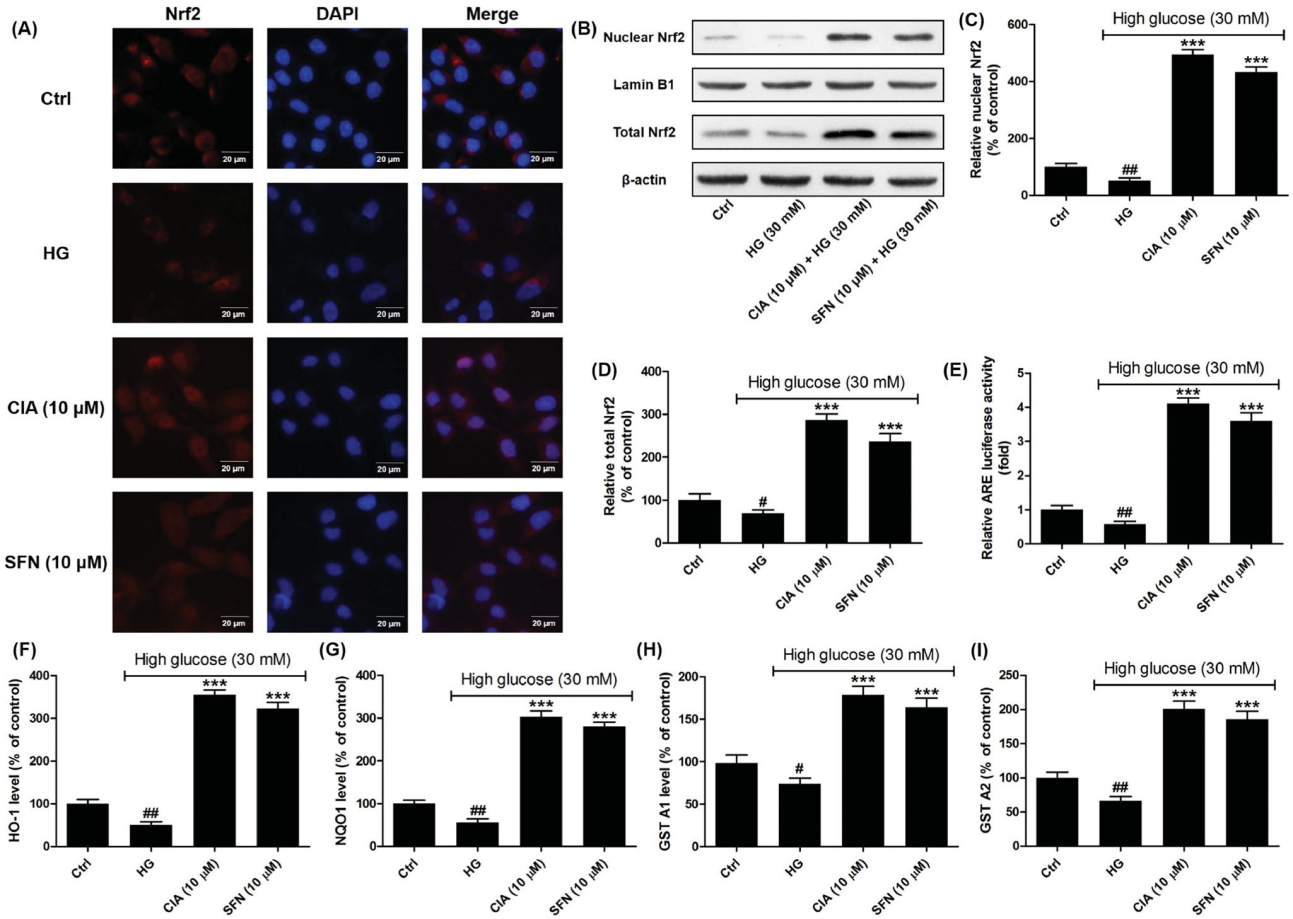
Effects of caffeoylisocitric acid on the activation of Nrf2 in mesangial cells under high glucose. (A) Immunofluorescence staining for Nrf2. (B) Western blot analysis for the expression of nuclear and total Nrf2. (C)-(D) Densitometric analysis for the expression of nuclear and total Nrf2. (E) Relative ARE-luciferase activity. (F)-(I) ELISA assays for the levels of HO-1, NQO1, GST A1 and A2. *n* = 3, ^#^*P* < 0.05 and ^##^*P* < 0.01 *vs* control group, ***P < 0.001 *vs* high glucose group.

From Figure 3 the localisation of Nrf2 in mesangical cells can be visualised through immunofluorescence staining. It was observed Nrf2 in mesangial cells exposed to caffeoylisocitric acid translocated into nuclei compared to the high glucose group, which was similar to the cells treated by sulforaphane. Western blot together with densitometric analysis disclosed caffeoylisocitric acid increased the expression of nuclear and total Nrf2, which indicated it could stabilise Nrf2 against the degradation and promote its nuclear translocation. The results from dual luciferase reporter assay showed caffeoylisocitric acid could enhance the transcription of ARE, which indicated the capacity of Nrf2 binding to ARE was elevated. In addition, as the detoxifying and antioxidant phase II enzymes, HO-1, NQO1, GST A1 and A2 were detected to further reveal the activation of Nrf2. The results showed high glucose reduced the levels of these enzymes in mesangial cells, whereas caffeoylisocitric acid enhanced the expression of these proteins similar to sulforaphane. Collectively these results unravelled caffeoylisocitric acid activated Nrf2 in mesangial cells under high glucose.

### 3.3. Caffeoylisocitric acid ameliorates oxidative stress in mesangial cells under high glucose

**Figure 4.**
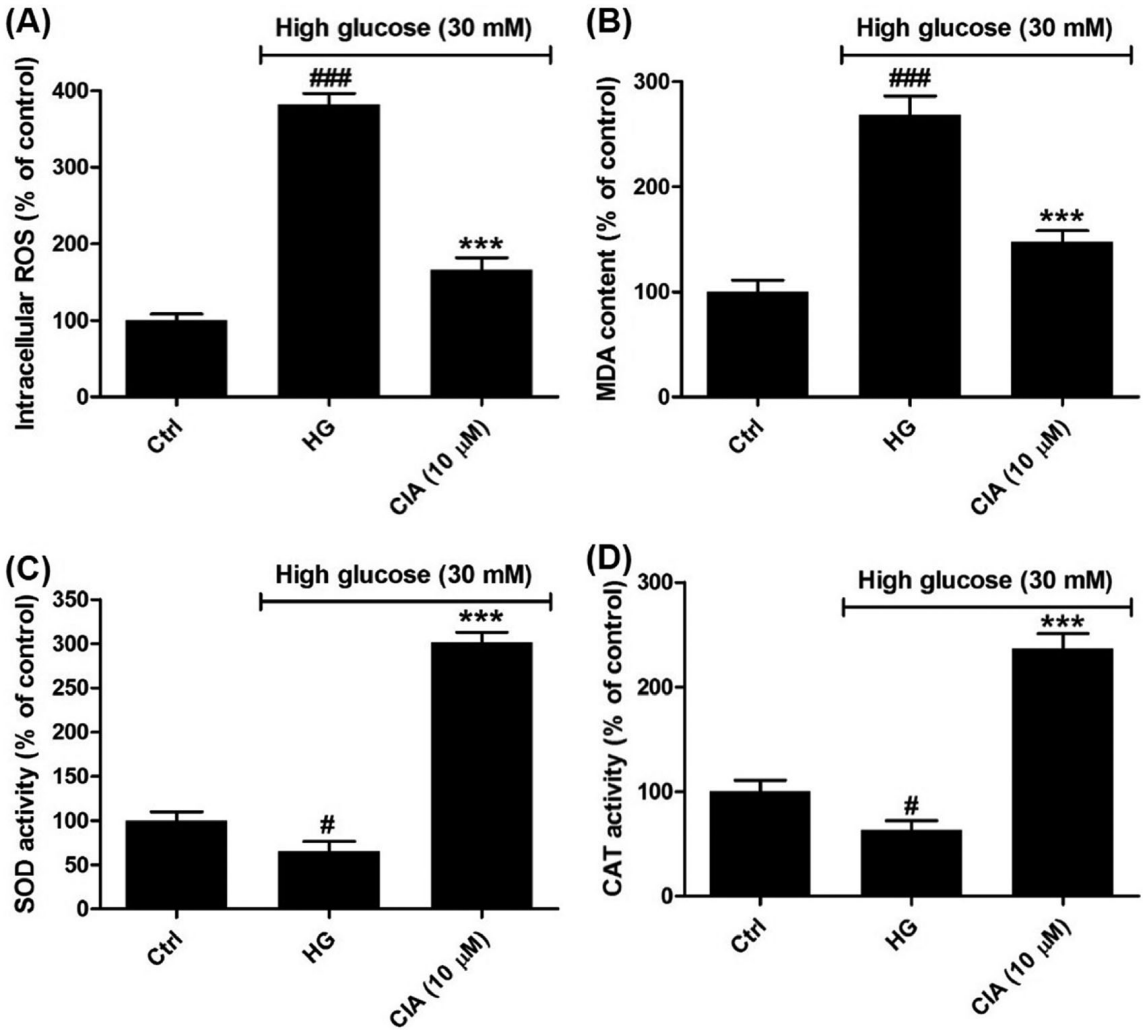
Effects of caffeoylisocitric acid on the oxidative stress induced by high glucose in mesangial cells. (A) Intracellular ROS levels. (B) MDA content. (C)-(D) SOD and CAT activity. *n* = 3, ^#^*P* < 0.05 and ^###^*P* < 0.001 *vs* control group, ***P < 0.001 *vs* high glucose group.

In diabetic kidney, ROS are overproduced due to the abnormal mentalism of glucose, which afford the amplification of glucose signalling^32^. Among the members of ROS, superoxide anion is generated from the activation of NADPH oxidase and mitochondrial electron transport chain^33^. SOD can convert superoxide to hydrogen peroxide, which can be further converted to water by CAT^34^. In Figure 4, high glucose has led to the overproduction of ROS (382.0 ± 14.1%) compared to control group. However, in the presence of caffeoylisocitric acid, the excessive ROS was markedly reduced (165.8 ± 15.9%). Meanwhile, as the products of lipid peroxidation, the content of MDA was elevated by high glucose (268.2 ± 18.1%), which was significantly decreased by caffeoylisocitric acid (147.3 ± 10.8%). In addition, though high glucose resulted in the decline for the activity of both SOD (65.2 ± 11.1%) and CAT (63.1 ± 9.2%), whereas the activity was reversed in the presence caffeoylisocitric acid (301.4 ± 11.5% for SOD and 236.5 ± 14.3% for CAT). These evidences indicated caffeoylisocitric acid inhibited oxidative stress induced by high glucose in mesangial cells.

### 3.4. Caffeoylisocitric acid attenuates inflammation in mesangial cells under high glucose

**Figure 5.**
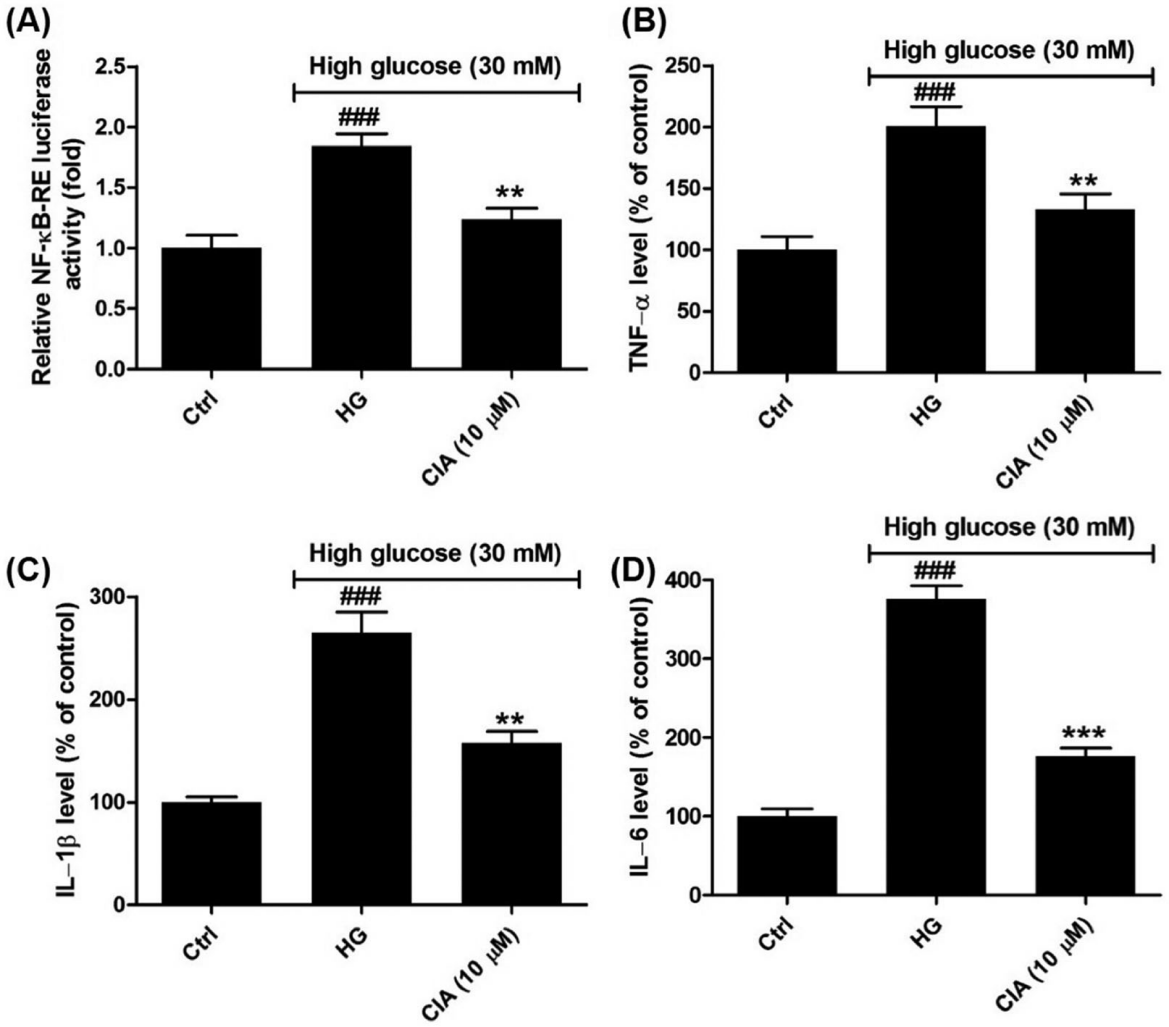
Effects of caffeoylisocitric acid on the inflammation induced by high glucose in mesangial cells. (A) Relative NK-κB-RE luciferase activity. (B)-(D) Relative levels of pro-inflammatory cytokines including TNF-α, IL-1β and IL-6. *n* = 3, ^###^*P* < 0.001 *vs* control group, **P < 0.01 and ***P < 0.001 *vs* high glucose group.

As shown in Figure 5, the capacity for transcriptional regulation of NF-κB was explored using dual luciferase reporter system. The results showed high glucose had stimulated the activity of NF-κB in mesangial cells compared to control group (*P* < 0.001), which was blocked after exposed to indicated caffeoylisocitric acid (*P* < 0.01). At the same time, the levels of downstream pro-inflammatory cytokines including TNF-α, IL-1β and IL-6 were determined by ELISA. It was observed that high glucose induced the excessive secretion of TNF-α (200.8 ± 15.8%), IL-1β (265.0 ± 20.1%) and IL-6 (375.9 ± 16.5%) in contrast to control group. However, in the presence of caffeoylisocitric acid, the synthesis of TNF-α, IL-1β and IL-6 was reduced to (132.8 ± 12.7%), (157.6 ± 11.3%) and (176.5 ± 10.2%), respectively. These observations implied caffeoylisocitric acid ameliorated inflammation resulting from high glucose in mesangial cells.

### 3.5. Caffeoylisocitric acid alleviates accumulation of ECM in mesangial cells under high glucose

**Figure 6.**
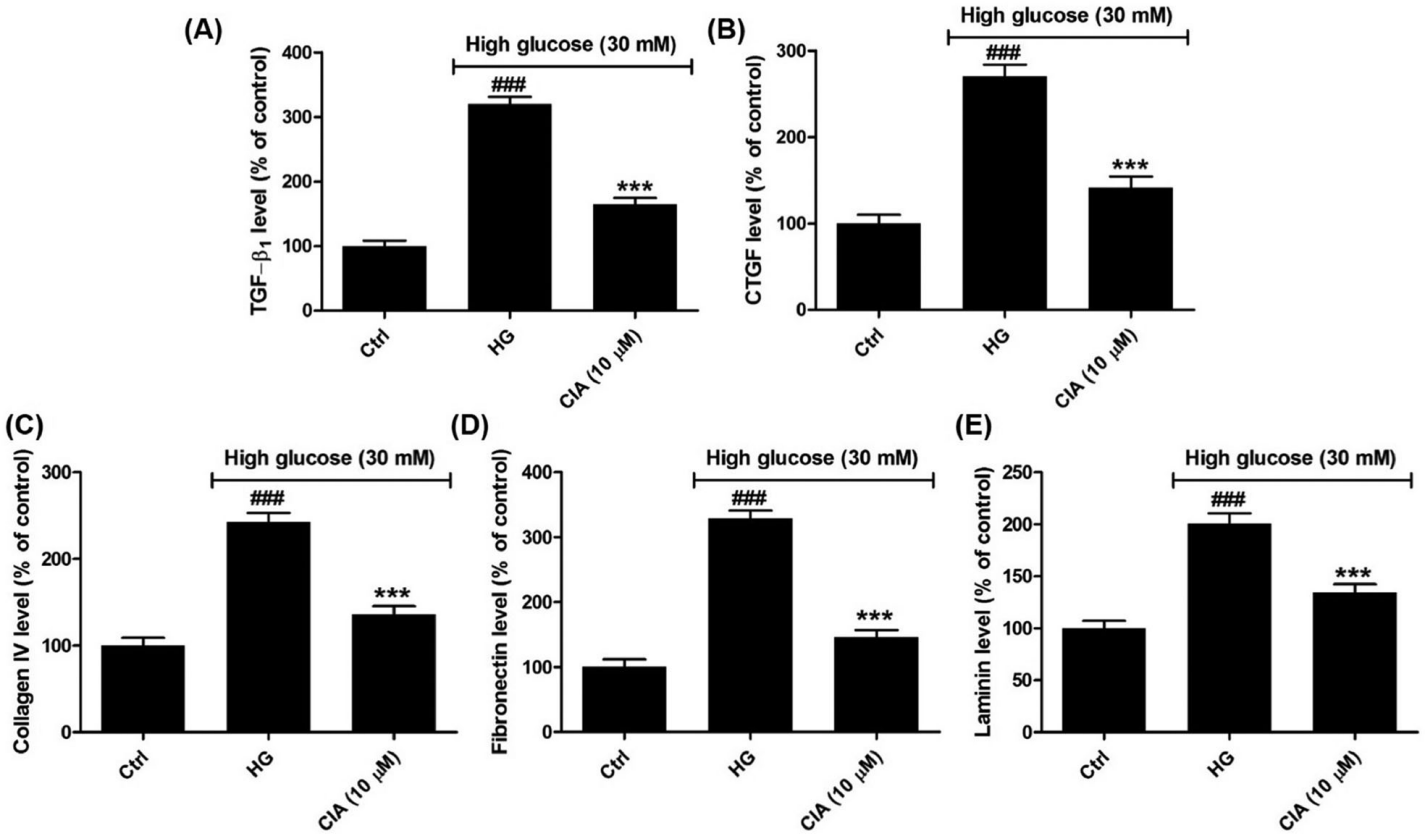
Effects of caffeoylisocitric acid on the accumulation of ECM induced by high glucose in mesangial cells. (A)-(B) Secretion of TGF-β_1_ and CTGF in mesangial cells. (C)–(E) Synthesis of ECM proteins including collagein IV, fibronectin and laminin. *n* = 3, ^###^*P* < 0.001 *vs* control group, ***P < 0.001 *vs* high glucose group.

Fibrosis is defined as the process of excessive ECM proteins accumulation^35^. Among the cytokines and growth factors, TGF-β_1_ is prominent in the renal fibrosis since it can promote synthesis of ECM and inhibit its degradation^36^. As the downstream cytokine of TGF-β_1_, CTGF is induced by TGF-β_1_ and involved in the enhanced synthesis of ECM^37^. Herein (Figure 6), the results displayed the secretion of both TGF-β_1_ and CTGF was markedly elevated by high glucose as 320.0 ± 11.0% and 270.5 ± 17.4%, but caffeoylisocitric acid at 10 µM significantly blocked their synthesis (164.4 ± 9.7% for TGF-β_1_ and 141.0 ± 13.2% for CTGF. Further detection on the major constituents of ECM encompassing collagen IV, fibronectin and laminin disclosed in mesangial cells high glucose promoted synthesis of these proteins as 242.5 ± 10.6%, 328.6 ± 12.2% and 200.7 ± 9.8%, respectively. Nevertheless, following the addition of caffeoylisocitric acid, the overproduction of collagen IV, fibronectin and laminin has been remarkably reduced as 136.0 ± 9.6%, 146.5 ± 10.3% and 134.5 ± 7.9%, respectively. These facts demonstrated caffeoylisocitric acid suppressed the excessive secretion of ECM in mesangial cells under high glucose.

### 3.6. Caffeoylisocitric acid inactivates MAPK signalling pathway in mesangial cells under high glucose

**Figure 7.**
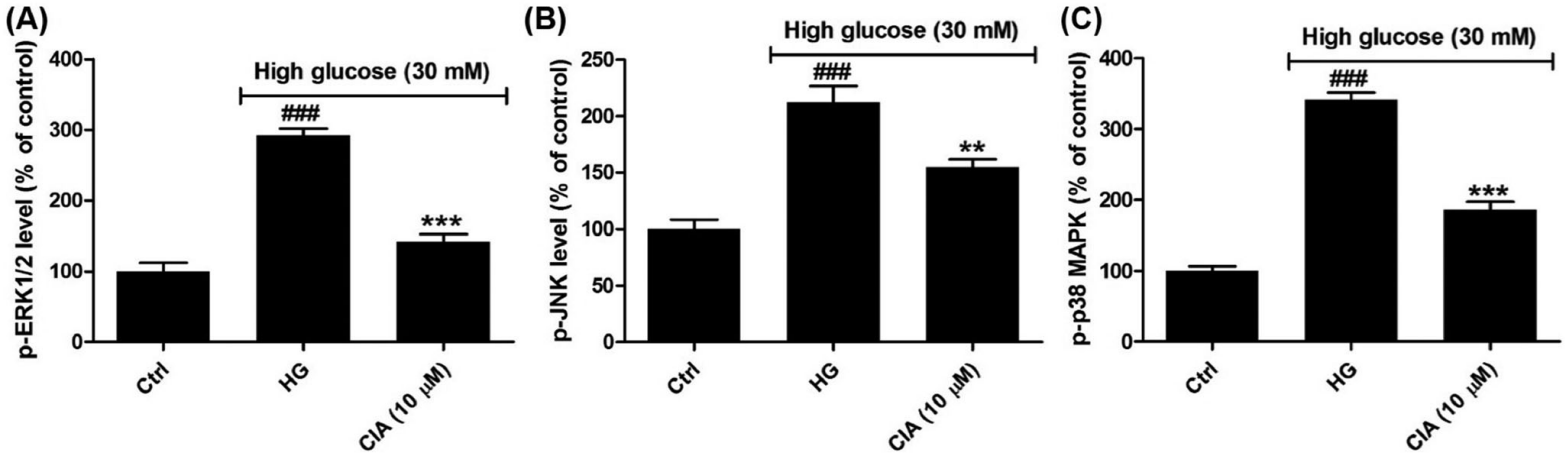
Effects of caffeoylisocitric acid on MAPK signalling pathway in mesangial cells under high glucose. (A)-(C) Levels of p-ERK1/2, p-JNK and p-p38 MAPK in mesangial cells. *n* = 3, ^###^*P* < 0.001 *vs* control group, **P < 0.01 and ***P < 0.001 *vs* high glucose group.

In the progress of DN, MAPK signalling has been the mediator of oxidative stress and promotes inflammation and accumulation of ECM through enhancing the expression of NF-κB and TGF-β_1_^3^^,^^4^. As the family of serine/threonine kinases, three subfamilies of MAPK have been identified and characterised as ERK1/2, JNK and p38 MAPK, which are activated through phosphorylation under the stimulation of high glucose in mesangial cells^38^. Herein the activated forms of these kinases were detected by ELISA. As shown in Figure 7, high glucose caused the increasing levels of the active forms of MAPK proteins including p-ERK1/2 (292.6 ± 9.6%), p-JNK (212.2 ± 14.5%) and p-p38 MAPK (341.3 ± 9.9%). However, in the presence of caffeoylisocitric acid at 10 µM, the activated p-ERK1/2, p-JNK and p-p38 MAPK were repressed as 141.5 ± 10.9%, 154.6 ± 7.2% and 185.2 ± 11.9%, respectively. These observations uncovered caffeoylisocitric acid inactivated the MAPK pathway.

### 3.7. Activation of Nrf2 is involved in the catabatic effects of caffeoylisocitric acid on mesangial cells under high glucose

**Figure 8.**
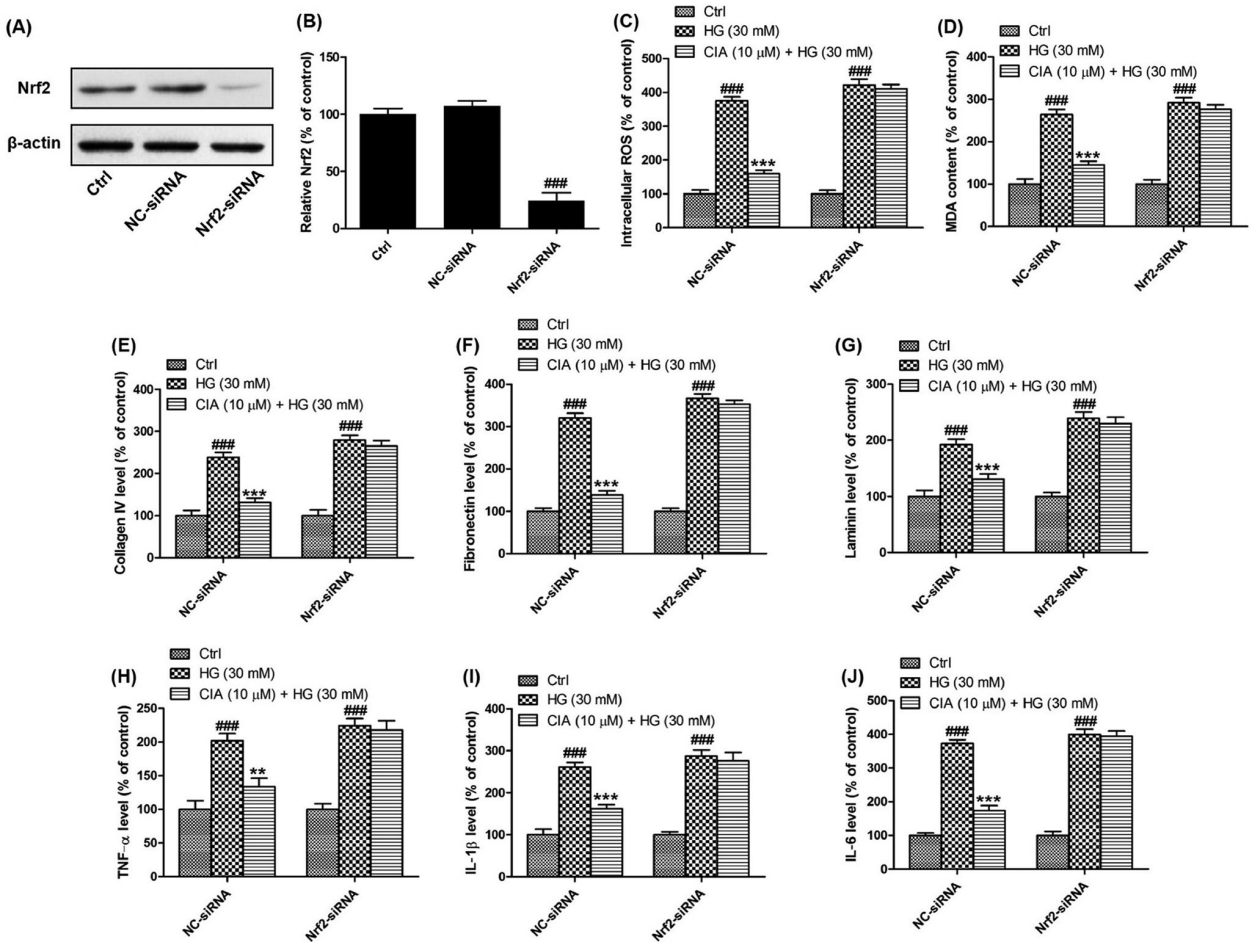
Activation of Nrf2 is involved in the effects of caffeoylisocitric acid in mesangial cells under high glucose. (A) Western blot analysis for the Nrf2 knockdown. (B) Densitometric analysis for the expression of Nrf2. (C)-(J) Intracellular ROS and MDA as well as extracellular collagen IV, fibronectin, laminin, TNF-α, IL-1β and IL-6 in mesangial cells transfected with Nrf2-siRNA or NC-siRNA exposed to caffeoylisocitric acid. *n* = 3, ^###^*P* < 0.001 *vs* control group, **P < 0.01 and ***P < 0.001 *vs* high glucose group.

To elucidate the role of Nrf2 in the effects of caffeoylisocitric acid, Nrf2 knockdown was implemented using siRNA interference (Figure 8). Western blot analysis together with densitometric analysis has validated the transfection with NC-siRNA or Nrf2-siRNA in mesangial cells was successfully attained. Then the redox status in mesangial cells was monitored. Compared to the cells transfected with NC-siRNA, increased ROS and MDA resulting from high glucose in the cells transfected with Nrf2-siRNA were not reduced by caffeoylisocitric acid. Meanwhile, though in mesangial cells transfected with NC-siRNA, excessive synthesis of collagen IV, fibronectin and laminin induced by high glucose was attenuated after exposed to caffeoylisocitric acid, it was not observed in cells transfected with Nrf2-siRNA. Furthermore, the pro-inflammatory cytokines including TNF-α, IL-1β and IL-6 were excessively secreted in mesangial cells transfected with NC-siRNA under high glucose, but were reversed in the presence of caffeoylisocitric acid. However, after transfected with Nrf2-siRNA, the secretion of pro-inflammatory cykines by mesangial cells couldn’t be affected by caffeoylisocitric acid obviously. From these results it can be derived activation of Nrf2 was closely associated with the effects of caffeoylisocitric acid.

### 3.8. Caffeoylisocitric acid interacts with Keap1 to activate Nrf2

**Figure 9.**
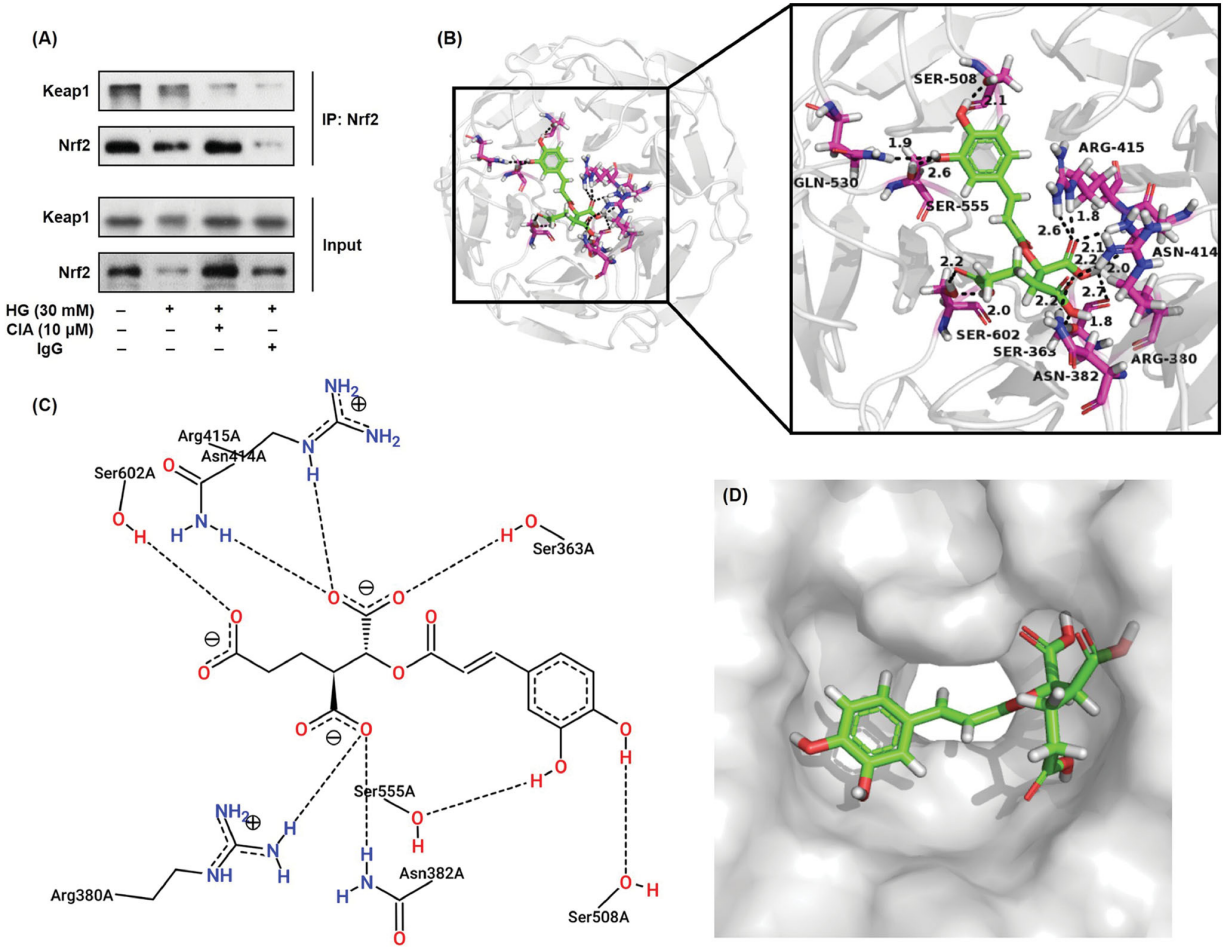
Interaction between Keap1 and caffeoylisocitric acid to activate Nrf2. (A) Co-immunoprecipitation assay for Nrf2. (B)-(D) Molecular docking for Kelch domain and caffeoylisocitric acid.

To elucidate the underlying mechanisms for caffeoylisocitric acid activating Nrf2 in mesangial cells, immunoprecipitation assay was performed firstly. As shown in Figure 9, when Nrf2 was precipitated using its primary antibody, Keap1 was also pulled down in both control and high glucose groups. However, in the presence of caffeoylisocitric acid, Keap1 co-precipitated with Nrf2 in mesangial cells was reduced. These results uncovered caffeoylisocitric acid could disrupt Keap1-Nrf2 interaction in mesangial cells. Further molecular docking has given the detailed interaction between Keap1 and caffeoylisocitric acid. The results showed caffeoylisocitric acid could enter the Nrf2 binding cavity in Kelch domain of Keap1 to form the complex through the hydrogen bonds with residues comprising Ser363 (1.8 and 2.7 Å), Arg380 (2.2 Å), Asn382 (2.2 Å), Asn414 (2.0 and 2.1 Å), Arg415 (1.8 and 2.6 Å), Ser508 (2.1 Å), Gln530 (1.9 Å), Ser555 (2.6 Å), and Ser602 (2.0 and 2.2 Å). In addition, from the 2D interaction diagram it can be found electrostatic interaction and Van der Waals’ force were also involved in the formation of Keap1-caffeoylisocitric acid complex. According to the molecular dynamics stimulations and free energy calculations, the Nrf2 binding cavity in Kelch domain of Keap1 can be divided into six subpockets (P1–P6)^26^. Small molecules interacting with the key residues in these subpockets will prevent the formation of Keap1-Nrf2 complex and activate Nrf2^39^. Herein, molecular docking results showed some residues interacting with caffeoylisocitric acid in the Kelch domain locate P1 (Arg415 and Ser508), P2 (Ser363, Arg380 and Asn414), P3 (Ser555 and Ser602) and P4 (Gln530). In addition, electrophiles can induce the conformation changes of Keap1 via interaction with its key cysteine residues in both BTB and IVR domains and result in the activation of Nrf2^40^. Molecules with α,β-unsaturated carbonyl moieties are typical electrophiles as Michael receptors. And the compounds with catechol moieties also belong to Michael receptors because the catechol can readily be oxidised to electrophilic 1,2-benzoquinone. These Michael receptors are usually considered as soft Lewis acids and react with the critical cysteine thiolate (soft Lewis base) in Keap1 through Michael addition^41^. In the structure of caffeoylisocitric acid, α,β-unsaturated carbonyl and catechol moieties can be found, which may be involved in the activation of Nrf2 through the covalent modification. On the whole, these results implicated caffeoylisocitric acid could interact with Keap1 to disrupt the formation of Keap1-Nrf2 complex and activate Nrf2.

## 5. Conclusion

In conclusion, we have identified caffeoylisocitric acid as a natural Nrf2 activator in glomerular mesangial cells. In addition to inhibition of the self-limited proliferation, caffeoylisocitric acid at 10 µM also activates Nrf2 in mesangial cells to attenuate oxidative stress, inflammation and accumulation of ECM resulting from high glucose via inactivating MAPK signalling pathway. The activation of Nrf2 by caffeoylisocitric acid is implemented through disrupting the interaction between Keap1 and Nrf2. These results provide evidences for caffeoylisocitric acid to be a simple lead for the discovery of novel Nrf2 activator targeting DN.
